# Effect of the Early Start Denver Model on Children With Autism Spectrum Disorder Syndrome of Different Traditional Chinese Medicine Types in Northeast China

**DOI:** 10.3389/fped.2022.851109

**Published:** 2022-03-29

**Authors:** Lili Wang, Junyan Feng, Yu Zhang, Tiantian Wang

**Affiliations:** Developmental Behavioral Pediatrics, The First Hospital of Jilin University, Jilin, China

**Keywords:** autism spectrum disorder, early start denver model, traditional Chinese medicine types, kidney jing deficiency, liver qi stagnation

## Abstract

**Background:**

The clinical presentation of children with autism spectrum disorder (ASD) is heterogeneous, and there are little data available on the treatment of children with different types of ASD. We sought to explore which traditional Chinese medicine (TCM) syndrome type was more effective for children with ASD after 3 months of Early Denver Model intervention and to analyze the reasons for its efficacy from the perspective of TCM.

**Methods:**

This was a retrospective study. The subjects were children with ASD who were first diagnosed at the Developmental Behavioral Pediatrics, the First Hospital of Jilin University, between December 2018 and September 2019. Eighty-nine children were divided into a kidney jing deficiency group, a liver qi stagnation group, and a group with deficiency of both the heart and spleen.

**Results:**

After treatment, the total Autism Behavior Checklist (ABC), Autism Treatment Evaluation Checklist, and Childhood Autism Rating Scale scores were significantly reduced in the three groups (*p* < 0.05) compared to before treatment. Significant improvements were seen in all five domains of the Griffiths Development Scales-Chinese version in the LQ group (*p* < 0.05). After intervention, the LQ group showed greater improvements compared to the other two groups in the language, eye–hand coordination, body and object use, social and self-help, and total ABC scores.

**Conclusion:**

Our study showed that Early Denver Model intervention is effective in the treatment of three syndrome types of children with ASD, with the LQ group experiencing the most significant effects.

## Introduction

Autism spectrum disorder (ASD) is a heterogeneous group of neurodevelopmental conditions characterized by the presence of impaired social communication and reciprocity and a restricted and stereotyped pattern of behaviors and interests. In the last few decades, the prevalence of ASD has increased dramatically, appearing as a sort of “epidemic,” affecting 1 in 59 children in the United States and significantly influencing the quality of life of children and their families because of the core developmental disability and associated medical and behavioral symptoms (1). In Jilin City of China, 77 cases of autism were identified from a total population of 7258, equating to a prevalence of 108 per 10,000 (2). Effective therapies for ASD core symptoms have not yet been established. Evidence-based first-line treatments are represented by behavioral therapies. The Early Start Denver (ESDM) model is an intervention for pre-school–aged children, which incorporates behavioral, developmental, and relationship-based strategies within a naturalistic teaching framework (3). The ESDM is specifically designed for children aged 12–60 months and is a developmental- and relationship-focused intervention that incorporates techniques designed to foster positive relationships between parent and child and to increase the child’s motivation to engage in social interactions (4, 5). At present, ESDM has been applied in the intervention of children with ASD and achieved satisfactory clinical effects (6, 7). However, there are still some children with ESDM intervention whose efficacy is not significant, which is related to a variety of factors, such as intervention scenarios and differences in educational concepts. Due to the heterogeneity of clinical symptoms, we observed that the clinical manifestations of children with ASD of the same severity were inconsistent. Some children were irritable, while others avoided eye contact. This variation may be associated with different subtypes, yet there are no relevant clinical subtypes of ASD. From the point of view of traditional Chinese medicine (TCM), the different types of syndrome differentiation represent reasons for the inconsistent clinical symptoms and manifestations of these children. At present, there is no study that has observed the therapeutic effect of ESDM by TCM syndrome–differentiation analysis in children with ASD. The hypotheses of this study are as follows: first, children with different types of ASD will experience different therapeutic effects of ESDM; second, for children with autism, a single treatment method is not enough, and more comprehensive therapy plans are needed for joint treatment, which may have a better effect.

## Materials and Methods

### Ethics Statement

The study protocol was approved by the ethics committee of the First Hospital of Jilin University (approval no. 20170107) and was registered with the Chinese Clinical Trial Register Center (registration no. ChiCTR1800019702) on November 24, 2018. The parents of the study participants provided written informed consent for inclusion in this study before enrollment.

### Study Participants

This was a retrospective study. Participants of the study were children with ASD who were first diagnosed at the Developmental Behavioral Pediatrics, the First Hospital of Jilin University, between December 2018 and September 2019. Inclusion criteria were as follows: first, mild to moderate ASD Children. The children were diagnosed by a multidisciplinary team following the American Psychiatric Association Diagnostic and Statistical Manual of Mental Disorders (DSM)-5 criteria (8); second, the participant was aged 24–60 months; and, third, the parents/caregivers understood the content of the study and agreed to participate in it, to receive 3 months of ESDM intervention and 10 sessions of parent skills training after having a conversation with the researchers, and to sign the informed consent form prior to enrollment. Conversely, individuals with Rett syndrome, fragile X syndrome, Angelman syndrome, Prader-Willi syndrome, tuberous sclerosis, or another syndrome caused by known genetic defects or inherited metabolic diseases and those with brain injuries and physical or sensory disabilities were excluded. Parents who did not provide home videos as assigned 3 times across the 3 months also prompted the exclusion of their children from this study. All potential participants were selected based on the criteria listed above.

### Study Protocol (Syndrome Differentiation Type)

The pathologic involvement of ASD is in the brain (in Chinese medicine, the brain usually refers to brain dysfunction), relating to the heart, liver, spleen, and kidney sin TCM theory (9). The concept of syndromes (zhengs) is unique to Chinese medicine. Syndromes are identifiable from a holistic understanding of a patient’s clinical presentation using the four Chinese medicine diagnostic methods: observation, listening/smelling, questioning, and pulse analyses. At present, there is no standardized of TCM syndrome types for ASD children, and the reports are inconsistent. In this study, TCM syndrome types for ASD children were classified according to the clinical manifestations, and based on Table 1 (10). When ASD children have multiple clinical manifestations, the most important clinical manifestation is used as the basis of TCM syndrome types assessment. A total of 89 ASD children were classified by TCM, each child were performed by two experienced TCM attending physicians independently. If the results are inconsistent, the disagreements were resolved through discussions with another senior physician.

**TABLE 1:** TCM syndrome differentiation of ASD.

Type	Clinical manifestation
Kidney jing deficiency	Low intelligence, sluggish expression, insensitivity, can hear but not respond
	A whitish tongue; deep, thready pulse
Liver qi stagnation	Firing of the heart and liver, impulsivity, quick temper, rash actions, red face, thirsty
	Red tongue or red tip of the tongue, thin and yellow tongue, a wiry pulse
Deficiency of both the heart and spleen	Speaking less, speaking mistakenly, not speaking, making no acknowledgment of relatives or strangers, apathy, no willingness to participate in social communication, can hear but does not respond, speaks repetitively, words are hard to understand
	Whitish tongue, thready pulse

#### Kidney Jing Deficiency

From the perspective of TCM, the process of development involves the gradual filling of the kidney qi. When the kidney qi is deficient, it will affect the growth of children. The kidney qi rules over long-term memory, so a kidney jing deficiency will result in poor mental development. In a personal translation and commentary of the texts of Wu et al. from the Qing dynasty, Fredes (11) stated that a lack of communication in small children is related to impaired heart qi, allowed by insufficient kidney qi, inherited from parents with weak qi and blood. This supports the idea of a genetic origin of the condition. Clinical manifestations of a kidney jing deficiency include low intelligence; sluggish expression; insensitivity; the ability to hear but not respond; a whitish tongue; and a deep, thready pulse. For treatment, it is recommended to invigorate the kidney, replenish the essence, nourish the liver, and strong bones.

#### Liver Qi Stagnation

The liver is an unyielding viscus organ, storing blood and governing tendons. The liver controls activities, stores the ethereal soul, and corresponds to anger in emotion and shouting in sound. Additionally, the liver advocates dredging, when the liver’s dredging function is normal, the person’s qi is smooth, and they are in a happy mood. However, if liver function is lost, a person’s emotions will be affected and they will appear uninterested in talking or unhappy. Autisms are often rejected by their parents, teachers, or peers because of their problematic behavior. Children with autism exist in a state of poor mood for a long time, will appear internal fire, irritability, insomnia, and other symptoms, which can affect the overall development of the child. At the same time, the liver is tied to the eyes, and the function of the liver can also be reflected in the activity of the eyes. Children with autism exhibit a lack of eye contact or active avoidance of eye contact, which can also be considered to be closely related to reduced liver function. Clinical manifestations of liver qi stagnation include heat in the heart and liver, impulsivity, a quick temper, rash actions, a red face and thirst, a red tongue or red tip of the tongue, a thin and yellow tongue, and a wiry pulse, For treatment, it is recommended to soothe the liver and resolve depression.

#### Deficiency of Both the Heart and Spleen

The heart is the master of the zang-fu, which governs the blood, harbors the spirit, and controls mental and emotional activities. The existence of sufficient heart-yin and heart-blood moisten and nourish the spirit and keep it at peace. Balance in the heart is another key element because a heart-blood or -yin deficiency, as well as heart fire, will lead to abnormal psychological activity of the spiritual consciousness and thinking or a reluctance to communicate, manifested by lethargy and quietness, fidgety restlessness, or aggressive behaviors. The spleen stores an individual’s intentions, attention, and intelligence and corresponds to thinking in cognition. The nature of the spleen is quiet. Clinical manifestations of a deficiency in both the heart and spleen include speaking less, speaking mistakenly, not speaking, making no acknowledgment of relatives or strangers, apathy, an unwillingness to participate in social communication, showing the ability to hear but not respond, speaking repetitively, speaking words that are hard to understand, a whitish tongue, and a thready pulse. For treatment, it is recommended to invigorate the spleen and nourish the heart.

### Sample Size Calculation

According to previous data published by Li et al. (12), the main measurement indicator, the Autism Behavior Checklist (ABC), was decreased by 15 points to be effective, and was set as unilateral α = 0.05, β = 0.2. The sample size was calculated based on the following equation: n = (Za/2 + Zβ)2⋅(σ12 + σ22)/δ2. After calculation, the sample size for each group was 11 cases, and we estimated a 20% dropout rate, so the final sample size was 14 cases per group. A total of 42 subjects were required.

### Intervention

A total of 116 subjects signed the informed consent form to participate in this study; they were required to fill a demographic information sheet (including age, gender, parents’ age, financial income, and parents’ education level). Finally, 89 children were enrolled, divided into the kidney jing deficiency group (KJ group, *n* = 17), liver qi stagnation group (LQ group, *n* = 46), and the deficiency of both the heart and spleen group (HS group, *n* = 26) based on TCM syndrome differentiation. The three groups received intensive training in ESDM for 3 months, and the intervention was conducted by therapists trained in ESDM in our department. The intervention time was 2 h per day,6 days per week. The intervention was centered on the children, and the children’s social skills, comprehension and expressive communication, joint attention, imitation, cognition, gross and fine motor skills, and self-care abilities were improved through games. In addition, parents of children received 10 sessions of parent skills training, once a week for 3.5 h. This training was divided into two areas. First, there was a theoretical part, for a total of 10 sessions, covering how to seize the child’s attention, feel the fun of social conventions, establish back-and-forth interaction patterns, non-verbal communication, imitation, the antecedent–action–outcome relationship, joint attention, functional and symbolic games, and the development of speech and independent living. A theoretical knowledge assessment was conducted after class to monitor the learning quality. Second, there was the family video presentation, with two videos provided for training at home, each of which was3–5 min long, and which mainly focused on the intervention plan formulated by the therapist. The videos were reviewed by a therapist who has received advanced ESDM training. The main purposes of the videos were to ensure the quality and execution of parents’ training at home and to offer timely guidance and suggestions.

#### Baseline (T1) Assessments

At baseline, developmental and behavioral medical history, demographic factors, and family characteristics, including age, gender, maternal age, paternal age, and parents’ education degrees, were collected. Additionally, the following assessments were completed.

The Griffiths Development Scales-Chinese version (GDS-C) is a standardized developmental assessment tool used for children from birth to 8 years old in China. There are five domains (locomotion, personal–social, language, eye–hand coordination, and performance [A–E]) for toddlers < 2 years old and one more domain (practical reasoning [F]) for children aged 2–8 years old. The GDS-C was localized and validated from the extended and revised version of the Griffiths Mental Development Scales. A child’s developmental age is determined based on the norms for their numerical age, and developmental quotients (DQs) are calculated by the following equation: developmental age/chronological age × 100. DQs for domains have a mean of 100 points (standard deviation = 15 points).

The ABC is applicable to individuals >18 months old, with a total of 57 items, including 5domains of sensation, communication, somatic movement, language, and self-care, with a total possible score of 158 points. The higher the ABC score, the more severe the ASD symptoms; the score for normal children is <53 points (13).

The Childhood Autism Rating Scale (CARS) is applicable to children >2 years old. There are 15 items, each of which is scored 1–4 points to evaluate the social communication, behavior, emotion, and sensory perception abnormalities of children. The typically developing child should score <30 points, and the higher the total CARS score, the worse the ASD symptoms (14, 15).

Finally, the Autism Treatment Evaluation Checklist (ATEC) is applicable to children >2 years old, covering language, social ability, sensory, and behavioral factors. The total score ranges from 0 to 180 points and consists of four subscales: speech/language communication, sociability, sensory/cognitive awareness, and health/physical behaviors. The higher the ATEC score, the more severe the ASD symptoms (16).

#### Post-intervention (T2) Assessments

All measures were re-administered to the three groups of participants at 3 months.

#### Raters

All professionals who administrated the above mentioned assessments were trained and blinded to the group assignment of each participant.

### Statistical Analysis

All data collected were analyzed using the SPSS version 20.0 software program (IBM Corporation, Armonk, NY, United States). The normality of the data was analyzed using the Kolmogorov–Smirnov test.

Continuous data were means ± SDs or P50 (P25, P75) (i.e., median, 25th percentile, and 75th percentile measures), whereas categorical data were given as frequencies with percentages. Chi-square test were used to compare the distributions of demographic data among the three groups. Additionally, non-parametric tests, and one-way analysis of variance were used to compare the developmental outcomes and ASD symptoms of children in the three groups. An α value of ≤0.05 was accepted as the level of statistical significance.

## Results

### Subject Enrollment and Type Flowchart

**FIGURE 1:**
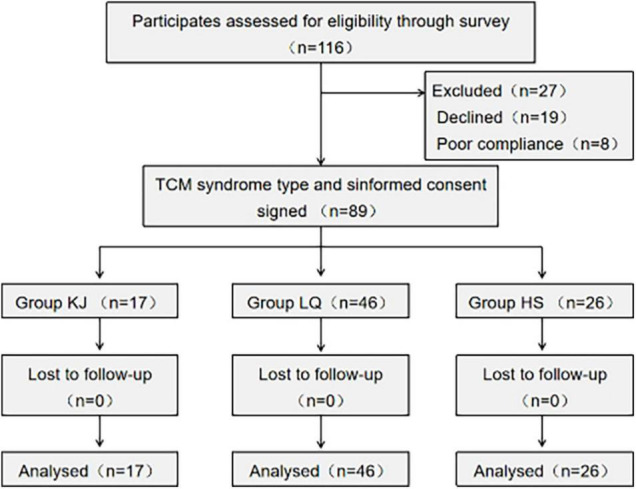
Flow diagram of the subject: screening, grouping, and intervention. KJ group, kidney jing deficiency group (*n* = 17); LQ group, Liver Qi stagnation group (*n* = 46); HS group, group with deficiency of both the heart and spleen (*n* = 26).

To identify the final group of participants, we set a protocol, including steps of enrollment and ASD types, to screen eligible and initial participants in accordance with the inclusion and exclusion criteria. Finally, 89 subjects were identified as eligible to join this clinical trial (Figure 1).

### Patient Demographic Characteristics

Collecting the baseline characteristics of patients was important for performing comparisons between the three groups. We needed to collect all possible demographic characteristics at baseline to describe subject homogeneity. Since age, gender, maternal age, paternal age, and parents’ education degrees are potential influencing factors in terms of the effect of intervention, these demographic characteristics were recorded in this study. There were 17 cases in the KJ group, including 15 boys and 2 girls, aged 25–58 months. There were 46 cases in the LQ group, including 40 boys and 6 girls, aged 24–50 months. Finally, there were 26 cases in the HS group, including 23 boys and 3 girls, aged 24–51 months. The main demographic characteristics of children, parents, and families in the KJ, LQ, and HS groups are presented in Table 2. Participants across the groups were well matched with respect to all demographic variables, although the LQ group included the most children among the three groups (51.7%).

**TABLE 2:** Patient demographic characteristics in the three groups.

Characteristics	KJ group	LQ group	HS group	χ^2^	*p*
			
*n*	17	46	26		
**Gender**				0.042	0.979
Male	15 (88%)	40 (87%)	23 (88%)		
Female	2 (12%)	6 (13%)	3 (12%)		
**Age, months**				1.560	0.458
24–36	2 (12%)	4 (9%)	3 (12%)		
37–48	12 (71%)	36 (78%)	18 (69%)		
49–60	3 (17%)	6 (13%)	5 (19%)		
**Education of the mother**				0.872	0.929
Primary	3 (17.6%)	7 (15.3%)	4 (15.4%)		
Secondary	12 (70.6%)	33 (71.7%)	19 (73.1%)		
Tertiary	2 (11.8%)	6 (13%)	3 (11.5%)		
**Education of the father**				0.334	0.988
Primary	3 (17.65%)	6 (13%)	4 (15.4%)		
Secondary	11 (64.7%)	33 (71.7%)	18 (69.2%)		
Tertiary	3 (17.65%)	7 (15.3%)	4 (15.4%)		
**Age of the mother, years**				1.297	0.523
16–24	0 (0)	1 (2%)	0 (0)		
25–34	16 (94%)	43 (94%)	24 (92%)		
35–44	1 (6%)	2 (4%)	2 (8%)		
**Age of the father, years**				1.486	0.476
16–24	1 (6%)	0 (0)	0 (0)		
25–34	14 (82%)	41 (89%)	23 (88%)		
35–44	2 (12%)	5 (11%)	3 (12%)		

### Comparison of the Clinical Efficacy of the Three Groups Between Before and After Treatment

There was no significant difference in GDS-C, ABC, CARS, and ATEC scores among the three groups before treatment (*p* > 0.05). After treatment, compared to before treatment, the total ABC, ATEC, and CARS scores were significantly reduced in all three groups (*p* < 0.05). Excluding the locomotor domain in the KJ group, there were significant improvements in other areas. In the HS group, there was no improvement in the language field, but significant improvements were observed in the other four fields. Significant improvements were also seen in all five domains of the GDS-C in the LQ group (*p* < 0.05). At T2, the DQs in the personal–social domain, language domain, eye–hand coordination domain, and performance domain of the GDS-C showed the most significant differences among the three groups (*p* = 0.011, *p* = 0.007, *p* = 0.002, and *p* = 0.007). In the locomotor domain, although the LQ group showed relatively obvious progress, there was no significant difference between the three groups (*p* = 0.062). See Table 3.

**TABLE 3:** Comparison of the GDS-C, ABC, CARS, and ATEC scores between T2 and T1.

	KJ group (*n* = 17)		LQ group (*n* = 46)		HS group (*n* = 26)			
	T1	T2	T1	T2	T1	T2	*p* value (among three groups T1)	*p* value (among three groups T2)
**GDS-C**								
A: Locomotor	72.6 ± 15.8	75.3 ± 15	78.2 ± 16.2	82.3 ± 15.1^#^	74.1 ± 15.7	74.5 ± 13.9^#^	0.499	0.062
B: Personal–social	50.5 ± 10.5	60.4 ± 14.2^#^	56.4 ± 19.7	72.2 ± 19.4^#^	50.2 ± 15.6	59.9 ± 19.3^#^	0.249	0.011*
C: Language	35(23,60)	54(39.5,62.5)^#^	40(35,59.3)	64(42.3,84)^#^	35(28,60)	35.5(26,65.3)	0.183	0.007*
D: Eye–hand coordination	57(41,63.5)	67(58,70.5)^#^	61.5(49.8,80)	80(65,86.5)^#^	61(50.5,74.8)	62.5(54.8,76.3)^#^	0.099	0.002*
E: Performance	60.4 ± 17.7	68.2 ± 16.8^#^	71.6 ± 20.5	83.6 ± 19.2^#^	62.3 ± 18.4	71.5 ± 22.2^#^	0.053	0.007*
**ABC**								
Sensory	7(3.5,12)	6(2.5,9)^#^	9(7,13)	8(4,9.3)^#^	8.5(5,12.5)	8(5,9.5)	0.139	0.237
Relating	15.7 ± 5.7	12.6 ± 5.3	15.8 ± 4.6	10.3 ± 5.6	16.3 ± 4.3	12.7 ± 6.1	0.878	0.138
Body and object use	10(4.5,15)	6(4,12.5)	10(7,16.3)	6.5(2,9.3)^#^	9.5(4,14.3)	7.5(4,10)^#^	0.413	0.252
Language	9.4 ± 5.2	8.8 ± 5.3	9.4 ± 4.8	8.8 ± 5.3^#^	10.9 ± 4.3	9.0 ± 6.2^#^	0.363	0.985
Social and self-help	10.6 ± 5.2	8.9 ± 4.4^#^	12.9 ± 5.1	8.3 ± 4.4^#^	12.6 ± 3.1	10.6 ± 4.5	0.219	0.114
Total score	53.3 ± 15.3	43.4 ± 13.9^#^	59.8 ± 14.4	40.4 ± 15.4^#^	58.6 ± 13.9	47.9 ± 13.9^#^	0.287	0.12
**ATEC**								
Speech/language communication	21(11,25)	17(9,24)^#^	14(9,22)	9(5.8,17.3)^#^	21(10,25)	15.5(7.8,24)^#^	0.139	0.019*
Sociability	15(11.5,19.5)	13(7.5,17.5)	14(7,20)	7(4,14.3)^#^	18.5(9,23.3)	13(7.3,17)^#^	0.204	0.035*
Sensory/cognitive awareness	16.6 ± 6.9	15.5 ± 7.0^#^	14.8 ± 8.2	11.2 ± 6.7^#^	17.4 ± 8.5	15.0 ± 8.3^#^	0.381	0.035*
Health/physical behaviors	11.6 ± 5.5	10.3 ± 4.4	11.4 ± 8.0	7.9 ± 6.2^#^	12.7 ± 8.8	9.3 ± 7.2^#^	0.83	0.374
Total score	64(43.5,74.5)	57(32.5,70.5)^#^	53(29.5,78.5)	36.5(20,57.5)^#^	70(35.8,85.8)	52.5(29.8,76)^#^	0.261	0.027*
**CARS**	32.0 ± 4.2	31.0 ± 4.8^#^	32.5 ± 3.8	29.3 ± 4.8^#^	33.1 ± 3.6	31.7 ± 3.5^#^	0.653	0.086

^#^T1 vs T2 (p < 0.05). *p < 0.05.

The change scores of DQ in the language domain and eye–hand coordination domain showed the most significant differences, with improvements of 15.5 and 12.8 points in the LQ group compared to 4.2 and 4.4 points in the HS group (*p* = 0.007 and *p* = 0.023). There was also no statistical difference in the ATEC change scores between the three groups. Also, though the ABC and CARS scores demonstrated a decreasing trend after the intervention, no significant difference was found between the three groups after intervention. However, the change scores for body/object use, social/self-help, and total ABC showed a significant difference among the three groups (*p* = 0.025, *p* = 0.029, and *p* = 0.002; Table 4).

**TABLE 4:** Comparison of ΔGDS-C, ΔABC, Δ CARS, and ΔATEC between the three groups after 3 months of treatment.

	KJ group (*n* = 17)	LQ group (*n* = 46)	HS group (*n* = 26)	*p* value (among three groups Δ)
		
	Δ	Δ	Δ	
**GDS-C**				
A: Locomotor	2.7 ± 13.5	4.1 ± 14.1	0.4 ± 12.7	0.534
B: Personal–social	10(3.5,18)	17(4.8,24.8)	8.5(4,17)	0.088
C: Language	12.0 ± 15.7	15.5 ± 14.9^b^	4.2 ± 11.2^c^	0.007*
D: Eye–hand coordination	9.7 ± 11.3	12.8 ± 13.1^b^	4.4 ± 10.5^c^	0.023*
E: Performance	7.8 ± 14.9	11.9 ± 16.2	9.2 ± 13.2	0.567
**ABC**				
Sensory	2.2 ± 4.3	3.1 ± 4.4	1.2 ± 4.5	0.203
Relating	3.1 ± 5.9	5.5 ± 6.2	3.6 ± 6.8	0.282
Body and object use	2.3 ± 7.3	5.6 ± 5.7^b^	2.0 ± 5.2	0.025*
Language	0.65 ± 6.2	0.5 ± 5.6	1.9 ± 5.5	0.588
Social and self-help	1.7 ± 4.5	4.6 ± 4.8^ab^	1.9 ± 4.8	0.029*
Total score	9.9 ± 12.5	19.3 ± 12.3^ab^	10.6 ± 9.1	0.002*
**ATEC**				
Speech/language communication	2.5 ± 4.5	3.9 ± 5.9	2.8 ± 4.9	0.564
Sociability	2.0 ± 6.4	4.7 ± 5.0	4.5 ± 7.4	0.281
Sensory/cognitive awareness	1.1 ± 6.4	3.6 ± 5.7	2.4 ± 5.9	0.327
Health/physical behaviors	1.3 ± 5.1	3.5 ± 5.8	3.4 ± 6.6	0.408
Total score	6.9 ± 14.1	15.6 ± 16.6	13.0 ± 20.3	0.220
**CARS**	1.0 ± 3.7	3.1 ± 4.4	1.4 ± 3.4	0.087

*^a^Between the KJ group and LQ group (p < 0.05).*

*^b^Between the LQ group and HS group (p < 0.05).*

*^c^Between the KJ group and HS group (p < 0.05).*

*p < 0.05.

## Discussion

Our study showed that ESDM can alleviate the core symptoms of children with ASD, but ASD has more heterogeneity and different efficacies. To our knowledge, this study is the first to observe the effects of ESDM intervention in children with ASD subtypes classified by TCM. The current study offers three main findings. First, the number of children in the LQ group was highest among the three groups. Second, ESDM was effective in the treatment of three syndrome types of children with ASD. Third, children in the LQ group performed better than those in the KJ group or the HS group under the ESDM intervention for 3 months.

The number of children in the LQ group was greatest (51.7%), which might be related to the climate characteristics and eating habits of northeast China, where a temperate monsoon climate reigns, but, because of the higher latitude, the warm summer is short and the cold winter is long. Here, food that can produce heat to help the consumer keep warm is given priority in the diet, and this tends to create an excess amount of “fire” that remains in the body. In the Guide to Clinical Practice with Medical Records: Synopses of Pediatrics, Ye Tianshi said, “the constitution of infants belongs to pure yang, so they are likely suffering from heat disease.” Thus, combined with the physiological characteristics of children, it is easy for an excess syndrome to form, which arises as the liver qi stagnation type in children with ASD. In this study, the participants with ASD were all permanent residents in northeast China, and their dietary habits were basically the same, involving food capable of the abovementioned effect.

The ESDM draws from teaching practices developed in the original Denver Model, such as relationship-based aspects of the therapist’s work with the child, using play as a foundation for learning, and using communication intervention principles from the field of communication science (3). Consistent with the results of other clinical studies, this study found that ESDM can effectively benefit children with ASD, and ESDM was effective in the treatment of three syndrome types of ASD. In addition, in the classification of TCM, the effect on children with the LQ type of ASD is more obvious. There are several reasons why this may be. First, in the intervention of ESDM, emotional problems of children with ASD can be easily observed clinically. The liver qi stagnation type of ASD often shows impulsivity, quick temper and rash actions. ESDM is based on children’s interests and carried out in the natural routine of daily play and care, which can well relieve the emotions of children with ASD and guide correct behaviors. Second, many studies (17–19) have shown that the parents of ASD children are under greater parenting stress. This kind of negative psychology of parents will also have a great negative impact on the family interaction mode and the intervention effects of children (20). Therefore, parents of ASD children need more information about ASD as well as emotional and social support (21). The main source of parental pressure is that children with ASD will have more behavioral problems due to difficulties in language communication and emotional regulation, while parents simultaneously lack corresponding skills, so they face great challenges in both nursing and intervention. This causes parents of children with ASD to lose control of their emotions and show anger when confronted by their children, leading to an increase in behavioral and emotional problems. When the emotion of children with ASD is relieved, the pressure of parents will be reduced, so that they can better interact with children. The liver qi stagnation type of ASD has prominent emotional problems. When the emotional problems are relieved, the progress of children can be seen immediately. Therefore, the effect of ESDM intervention on the improvement of children with the liver qi stagnation type of ASD is more obvious.

Syndrome differentiation aims to divide patients into several types according to their clinical symptoms and signs, which is essential for TCM. In TCM theory, the persistent emotional stimulus affects the function of the liver free flow and causes stagnation of the liver qi. Dysfunction of the liver free flow is often found in the early stage of ASD, which is characterized by mental depression and an apathetic expression. Depressed Liver qi transforms into fire, which is characterized by agitation and anger, a red face and eyes, constipation, and yellow urine. ASD children disregard other people, fail to look at each other, and avoid one’s eyes. Dysfunction in the liver free flow may also be observed because, from a TCM perspective, liver function is reflected in the activity of the eyes. From another point of view, this relates to the physiology and pathology of children on the one hand because a child’s “liver qi is not full” (The function of the liver is not fully developed) and, on the other hand, the “liver often has excess.” So, for liver regulation function in children, an understanding of the external environment is different from that in adults, and this is the main cause of mental behavior disorders in children. Therefore, the clinical manifestations of the liver qi stagnation type of ASD tend to be more centered in the areas of emotional problems and digestive problems (e.g., constipation and yellow urine). These problems are often more easily observed by parents and therapists, and the clinical symptoms may be improved through active intervention, which is more prominent in the score changes of ABC, CARS, and ATEC. On the other hand, the children in the KJ and HS groups show a deficiency syndrome. With clinical observations, we found that the pace of treatment and improvement of deficiency syndrome is lower than excess syndrome. The results of this study may be because the course of treatment was not long enough, in that 3 months of intervention did not produce a significant change in deficiency syndrome. In addition, the symptoms of deficiency syndrome (e.g., sluggish expressions, speaking less, and apathy) have not received enough attention because many parents think these are innate characteristics and timely intervention is not pursued, which is one of the reasons why deficiency syndrome is not easy to correct.

Some studies using TCM techniques other than behavioral therapies in ASD have already been performed and have reported beneficial effects, including acupuncture (10, 22, 23), Tuina (24–26), Qigong (27, 28), and herbal medicine (29). There is an inadequacy in the description of ASD in TCM in ancient documents. With the acknowledgment of relative disease, ASD exists in the grouping of “slow speaking,” “weak fetus,” and “wuchi” (“five retardations”). In TCM, zang-fu is a term for the organs of the human body. Many of the organ names are familiar terms, which refer not only to a physical organ but also to the energetic functions of that organ. Each organ relates to an emotional response, sensory organ, and soft tissue. Individuals with ASD experience difficulties with sensory integration. In the treatment of ASD, the four primary organ systems of concern are the heart, spleen, liver, and kidneys; these organ systems are associated with speech, taste, vision, and hearing. There is no single direct cause-and-effect relationship for ASD in Chinese medicine, though there are a set of cofactors that must be present. This forms the manifestation of different syndromes. TCM emphasizes performing treatment based on syndrome differentiation, which is the core technology that can embody the characteristics and advantages of TCM diagnosis and treatment. However, in the treatment of ASD, limited articles discussing treatment based on syndrome differentiation exist.

Based on the yin/yang theory, TCM views disease within the framework of energy balance. Therefore, the diet of children with ASD in northeast China should be adjusted mainly to reduce the intake of calories, and proper outdoor exercise should be prescribed to facilitate the release of residual fire from the body in order to achieve the goal of a balance between yin and yang in the body of children with ASD. Some studies have shown that acupuncture and massage have a certain clinical efficacy in improving gastrointestinal function and sleep by regulating the qi and blood, thereby restoring homeostasis and offering relief from many of the behavioral and regulatory symptoms commonly found in children with ASD (30–32). One of the principles of Chinese medicine emphasizes the connection and harmony of the body in which the external “skin” is closely related to the internal “organs.” Therefore, stimulation of the skin has been used as a way to stimulate internal organs to restore balance in the body. In future research, we will adopt dietary structure, exercise, acupuncture, and massage methods as clinical interventions for ASD, aiming to tend to the balance of yin and yang in children to improve their overall symptoms.

This research project has some limitations. For example, fewer measurement scales were used to measure the main clinical symptoms and accompanying symptoms in children with ASD. More comprehensive assessments including ADOS, ADIR, 6-Gastrointestinal Severity Index (6-GSI), Parenting Stress Index (PSI), Child Behavior Checklist (CBCL), and Children’s Sleep Habits Questionnaire (CSHQ) should be performed in our future investigations (33, 34). In the meantime, we hope to further explore the efficacy of traditional Chinese medicine treatment on autism, especially adolescent autism, by carrying out a larger research (35).

## Data Availability Statement

The raw data supporting the conclusions of this article will be made available by the authors, without undue reservation.

## Ethics Statement

The studies involving human participants were reviewed and approved by the Ethics Committee of The First Hospital of Jilin University. Written informed consent to participate in this study was provided by the participants’ legal guardian/next of kin.

## Author Contributions

LW and JF wrote the manuscript. YZ reviewed the literature and contributed to writing the manuscript. TW conceived the review and provided final approval of the version to be published. All authors contributed to the article and approved the submitted version.

## Conflict of Interest

The authors declare that the research was conducted in the absence of any commercial or financial relationships that could be construed as a potential conflict of interest.

## Publisher’s Note

All claims expressed in this article are solely those of the authors and do not necessarily represent those of their affiliated organizations, or those of the publisher, the editors and the reviewers. Any product that may be evaluated in this article, or claim that may be made by its manufacturer, is not guaranteed or endorsed by the publisher.
